# Dose-dependent dual effects of HDAC inhibitors on glial inflammatory response

**DOI:** 10.1038/s41598-025-96241-x

**Published:** 2025-04-10

**Authors:** Samantha Mancino, Mariaserena Boraso, Andrea Galmozzi, Melania Maria Serafini, Emma De Fabiani, Maurizio Crestani, Barbara Viviani

**Affiliations:** 1 Department of Pharmacological and Biomolecular Sciences “Rodolfo Paoletti”, Università Degli Studi Di Milano, Milan, Italy; 2 Departamento de Bioengenharia E Instituto de Bioengenharia E Biociências, Instituto Superior Técnico, Universidade de Lisboa, Lisbon, Portugal; 3 Associate Laboratory i4HB - Institute for Health and Bioeconomy, Instituto Superior Técnico, Universidade de Lisboa, Lisbon, Portugal; 4 Department of Biomolecular Chemistry School of Medicine and Public Health, University of Wisconsin-Madison, Madison, WI USA

**Keywords:** Neuroinflammation, Histone deacetylase inhibition, Trichostatin A, Suberoylanilide hydroxamic acid, MS-275, MC1568, Microarray analysis, Molecular biology, Neuroscience

## Abstract

Neuroinflammation is defined as a process that includes cellular responses designed to protect the central nervous system from external influences, and it initiates in cases of extreme deviations from homeostasis. While it serves a protective role, excessive immune activation can lead to the release of neurotoxic factors, worsening disease progression. Histone deacetylases (HDACs) have been shown to modulate the expression of inflammatory genes by remodeling chromatin through the process of histone deacetylation. HDAC inhibitors (HDACi) alter histone acetylation and affect the transcription of genes involved in inflammatory pathways, making them promising therapeutic tools for the modulation of a variety of inflammatory diseases. However, their use is limited due to non-specific targeting and contradictory results. This study aimed to reconcile conflicting results and share insights on relevant HDACi in the inflammatory response induced by lipopolysaccharide (LPS), considering different exposure scenarios, cellular models, and associated molecular pathways. Specifically, the study evaluated the dose-dependent effects of two broad-spectrum HDACi, Trichostatin A (TSA) and Suberoylanilide Hydroxamic Acid (SAHA, Vorinostat), alongside selective inhibitors—MS-275 (Entinostat, class I), and MC1568 (class II)—on the expression and release of pro- and anti-inflammatory cytokines. Broad-spectrum HDAC inhibitors TSA and SAHA exhibited dose-dependent modulation of LPS-induced cytokine release. Co-treatment with TSA and LPS enhanced pro-inflammatory cytokines (TNF-α, IL-1β) and decreased IL10 in a dose-dependent manner at lower doses (≤ 10 nM), while high concentrations (100 nM) induced the anti-inflammatory IL-10. Pre-treatment with TSA led to a reduction in TNF-α levels induced by LPS, without affecting IL-1β or IL-10 levels. In contrast, the presence of TSA in LPS-triggered alveolar macrophages resulted in a decline in the production of both pro- and anti-inflammatory cytokine, irrespective of the TSA concentration. SAHA exhibited dual effects, enhancing TNF-α and IL-1β at nanomolar levels but suppressing TNF-α at micromolar doses in co-treated glial cells with LPS. Class-selective inhibitors highlighted distinct HDAC roles on LPS modulation: MS-275 reduced, while MC1568 enhanced, TNF-α release, alongside varied IL-1β and IL-10 modulation. To better understand the dual effects of SAHA, transcriptomic analysis of glial cells was conducted in the presence of LPS and low and high SAHA concentrations (100 nM or 5 µM). This analysis revealed a dose-dependent alteration in gene expression and pathway enrichment associated with cytokine signaling and immune regulation (e.g., JAK-STAT). Altogether, these findings reveal insights on the subtle, dose- and context-dependent role of HDACi in modulating glia inflammation.

## Introduction

Marked inflammatory reactions in the central nervous system (CNS) contribute to neurodegenerative diseases^1^ and psychiatric disorders^2^. Epigenomic regulations, such as histone modifications^3^, underlie the induction and maintenance of the inflammatory state. Specifically, acetylation and deacetylation of lysine residues regulate gene expression by modifying chromatin conformation, ultimately affecting several cellular processes including inflammation^4^. These post-translational modifications are regulated by two classes of enzymes: histone acetyltransferases (HATs) and histone deacetylase (HDACs), respectively. HATs, add an acetyl group to lysine residues on the core histones, leading to a relaxed chromatin structure generally correlated with gene activation. Conversely, HDACs remove acetyl groups from lysine residues, stabilizing chromatin architecture and typically mediating transcriptional repression^5^. In neuronal cells, levels and activities of HATs and HDACs are finely balanced under normal conditions^6^. However, in a neurodegenerative state, histone acetylation homeostasis becomes greatly impaired, shifting towards hypoacetylation^6^. Consequently, the inhibition of HDACs has been explored to restore homeostatic conditions, highlighting the potential therapeutic value of HDAC inhibitors (HDACi)^7,8^.

Eighteen members of HDAC family have been identified in humans, divided into four classes: class I (HDACs 1, 2, 3, and 8), class IIA (HDACs 4, 5, 7, and 9), class IIB (HDACs 6 and 10), class III (Sirtuins 1 to 7), and class IV (HDAC 11)^9^. Class I HDACs are primarily located in the nucleus and maintain basic cellular functions such as regulation of gene transcription and cell proliferation^10^. They are mainly involved in innate immunity and they regulate inflammatory reactions, often implicated in acute inflammatory responses and highly expressed in immune cells, including glial cells^11^. Class II HDACs shuttle between the nucleus and cytoplasm, influencing signaling pathways that control cell differentiation, migration, and stress responses. These HDACs are mainly associated with adaptive immunity^12^ and are thought to modulate longer-term changes in immune cell function, such as the switch from pro-inflammatory to anti-inflammatory phenotypes^13^.

This different implication in the inflammatory process underscores the importance of investigating both class I and class II HDACs in the context of inflammatory regulation.

Suberoylanilide hydroxamic acid (SAHA) and Trichostatin A (TSA) are both nonselective small molecule inhibitors of the Zn (II)-dependent class I and class II HDACs^7^. TSA is a potent, broad-spectrum inhibitor affecting both class I and class II HDACs. SAHA, also known as Vorinostat, while also a broad-spectrum inhibitor, has been noted to exhibit specificity for most of the 11 metal-dependent HDAC isoforms^10,14^. TSA, commonly used in research, provides broad HDAC inhibition, whereas SAHA is used clinically for its more specific effects, particularly in treating cutaneous T-cell lymphoma^15^. SAHA is also undergoing clinical trials for other cancers^16^ and HIV^17^. In addition, specific class I and II HDAC inhibitors, such as MS-275 and MC1568, are relevant for inflammatory regulation^10^. MS-275 (Entinostat), a class I HDAC inhibitor, has shown promise in clinical trials for cancer treatments^18^, while MC1568, a class II inhibitor, is used in research and holds potential for modulating inflammatory pathways^19,20^. Together, these inhibitors allow us to explore the distinct and overlapping effects of class I and II HDAC inhibition on inflammation.

While SAHA is clinically approved for cancers, its use in neurodegenerative diseases remains mainly confined to the experimental stage^21^. This is primarily due to limited clinical evidence, toxicity concerns at higher doses required for anti-inflammatory effects narrowing its safe therapeutic window within the CNS, lack of HDAC isoform selectivity, which complicates achieving therapeutic CNS concentrations without systemic side effects^22^. Only recently it was tested in a trial for Alzheimer’s disease (NCT03056495) to establish its efficacy and determine the maximum tolerable dose. Other HDACi investigated in clinical trials for neurodegenerative disease are RG2833, a benzamide specific for HDAC3, and AMX0035 (NCT03533257), a pan-HDACi, investigated for Friedreich’s ataxia and Amyotrophic lateral sclerosis, Alzheimer’s disease, respectively^10,23,24^.

Current studies have yielded conflicting results regarding HDACi effects on resident immune cells such as microglia and astrocytes^25–28^, as well as on other cell types. For instance, HDACi treatment has been shown to reduce inflammation in primary mouse dendritic cells^29^, mixed CNS glia^11^, primary mouse microglia, hippocampal HT-22 cells^30^, and macrophages^31,32^. Conversely, some studies have reported an increase in inflammatory responses in mouse microglia cell lines^33^ and primary rat microglia^26,34^. The main challenge in interpreting these results is the variation in the experimental conditions across studies.

Given these uncertainties, in this study we aimed to investigate the dose-dependent modulatory effects of HDACi on inflammatory responses in CNS immune cell populations, focusing on the distinct roles of class I and class II HDACs. We hypothesized that the effects on inflammation depend not only on the class of HDAC inhibited but also on the treatment protocol (simultaneous vs. prior to inflammatory stimulus) and the specific cell population being targeted (microglia, astrocytes, macrophages). Addressing these variables is essential to clarifying the context-specific actions of HDACi, which may vary significantly across cell types and treatment paradigms. In the first part of this study, we compared broad-spectrum and class-selective HDAC inhibitors on the inflammatory responses of various cellular populations, under different treatment paradigms and doses. In the second part, by using transcriptomic analyses we highlighted the molecular pathways influenced by a specific HDAC inhibitor, SAHA, known for its clinical relevance and potential application in neurodegenerative diseases. These findings extend our knowledge on how HDACs regulate neuroinflammation and contribute to a more comprehensive understanding of HDACi as potential therapeutic agents.

## Results

### Dose-dependent effects of TSA on the expression of pro- and anti-inflammatory cytokines in LPS-co-stimulated mixed and glial cells

**Fig. 1:**
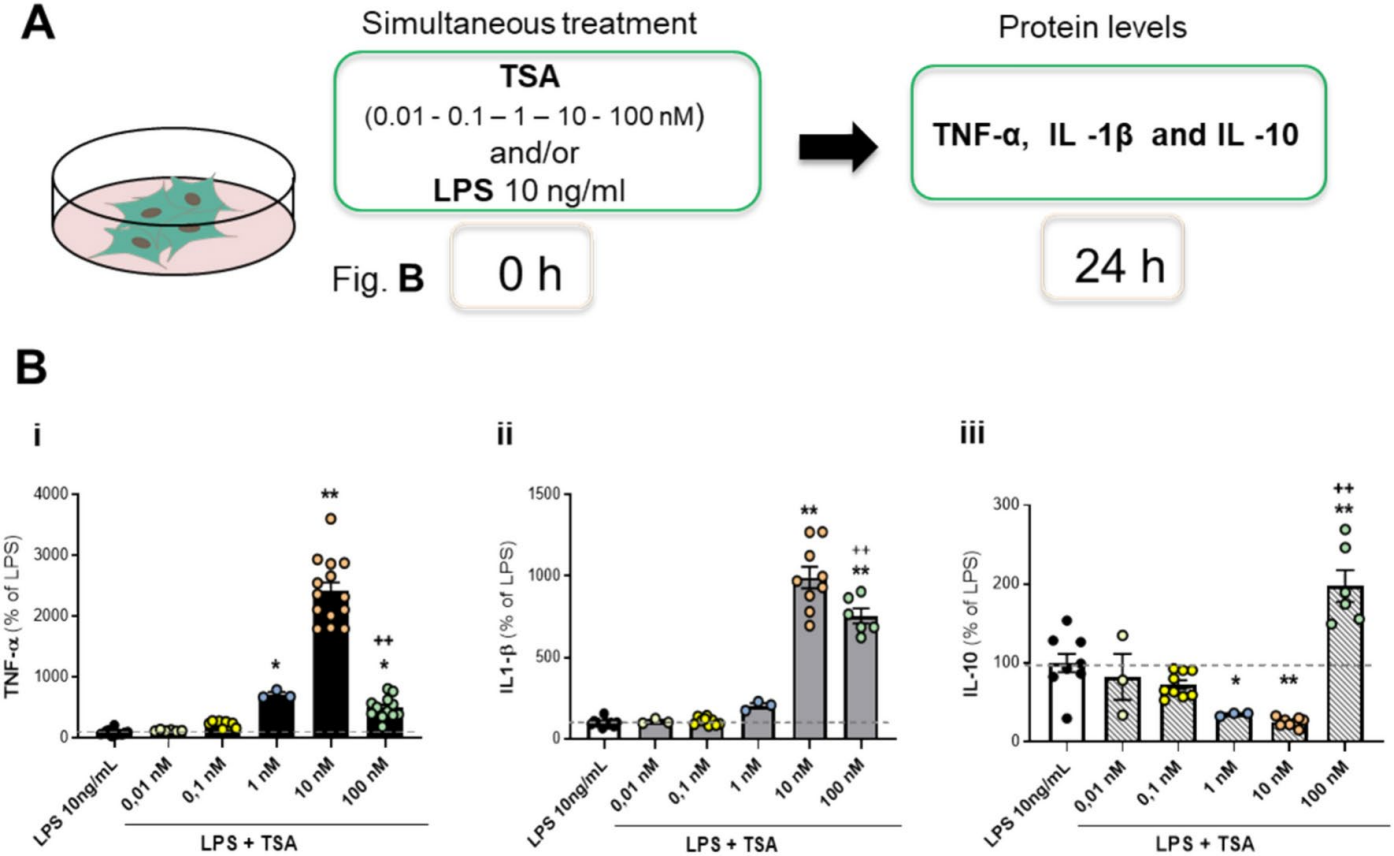
Effect on cytokine release after the simultaneous exposure to TSA and LPS in glial cells. (**A**) Experimental design for prolonged (24 h) exposure to TSA and/or LPS. (**B**) Evaluation of TNF-α (i), IL-1β (ii) and IL-10 (iii) release after 24 h exposure to 10 ng/ml LPS in absence or presence of different concentrations of TSA (0.01, 0.1, 1, 10, 100 nM). Results are expressed as a percentage of the LPS-treated control. Data are mean ± SEM of one to or four independent experiments in triplicate. Statistical analysis was conducted using one-way ANOVA followed by Tukey’s post hoc test.: *p < 0.05, **p < 0.01 LPS + TSA vs LPS; ^++^ *p* < 0.01 LPS + TSA 10 nM vs LPS + TSA 100 nM. Although not shown in the graph, the following significant differences were also observed: TNF-α (i): Comparisons of TSA 0.01, 0.1, and 1 nM vs. 10 nM (p < 0.01); IL-1β (ii): Comparisons of TSA 0.01, 0.1, and 1 nM vs. 10 and 100 nM (p < 0.01); IL-10 (iii): Comparisons of TSA 0.01, 0.1, and 1 nM vs. 100 nM (p < 0.01), and TSA 0.1 nM vs. 10 nM (p < 0.05).

To investigate how broad-spectrum inhibition of HDACs modulates the neuroinflammatory response in mixed glia in vitro, primary cultures of glial cells were simultaneously exposed to bacterial LPS (10 ng/ml) and increasing concentrations of TSA (0.01, 0.1, 1, 10, and 100 nM, respectively) for 24 h (Fig. 1A). The release of TNF-α and IL-1β (early phase pro-inflammatory cytokine) and IL-10 (late phase anti-inflammatory cytokine) in the culture medium was evaluated by a specific biological assay and ELISA, respectively (Fig. 1B). The selected concentration of LPS of 10 ng/ml elicited the highest cellular response without affecting their viability after 24 h of treatment (cell viability: control = 100% ± 10.4, LPS 10 ng/ml = 89.9% ± 10.3). Moreover, all tested concentrations of TSA, with or without LPS 10 ng/ml, did not affect glial cell viability (cell viability relative to the highest concentration of TSA: control = 100% ± 3.6, TSA 100 nM = 116.7 ± 4.6, LPS 10 ng/ml + TSA 100 nM = 86.7% ± 9.2). As shown in Fig. 1 B, TSA enhanced LPS-induced release of the pro-inflammatory cytokines, TNF-α (Fig. 1B, inset i) and IL–1β (Fig. 1B, inset ii). The effect was dose-dependent and maximal at the concentration of 10 nM TSA, whereas it became less pronounced at 100 nM TSA, (Fig. 1 B, insets i and ii). On the contrary, TSA reduced IL-10 production elicited by LPS treatment up to 10 nM (Fig. 1B, inset iii). However, at a concentration of 100 nM, TSA significantly reversed the trend and increased the production of the anti-inflammatory mediator IL-10 (Fig. 1B, inset iii). Treatment with TSA itself did not induce the release of TNF-α, IL-1β, or IL-10 (data not shown).

Mixed glial cells were also treated with 10 ng/ml LPS in the absence or presence of 10 nM TSA for 1 h (early phase; pro-inflammatory i.e. TNF-α and IL-1β) and 6 h (late phase; anti-inflammatory, i.e. IL-10) and the level of cytokine’s mRNA were evaluated by RT-PCR (Fig. S1). Accordingly, after 1 h, 10 nM TSA enhanced LPS-induced mRNA expression of pro-inflammatory cytokines, TNF-α and IL-1β, and significantly reduced LPS-induced mRNA of the anti-inflammatory cytokine IL-10 at 6 h (Fig S1).

Primary cultures of microglia or astrocytes were treated with 10 ng/ml LPS with or without 10 nM TSA for 24 h. At the end of the treatment, the release of TNF-α, IL-1β, and IL-10 in the culture medium was assayed. Consistent with the data obtained in the mixed glial population, 10 nM TSA significantly potentiated the LPS-induced release of the two pro-inflammatory cytokines TNF-α and IL-1β in both glial cell populations, whereas it inhibited the LPS-induced production of the anti-inflammatory cytokine IL-10 (Table 1).

**Table 1:**
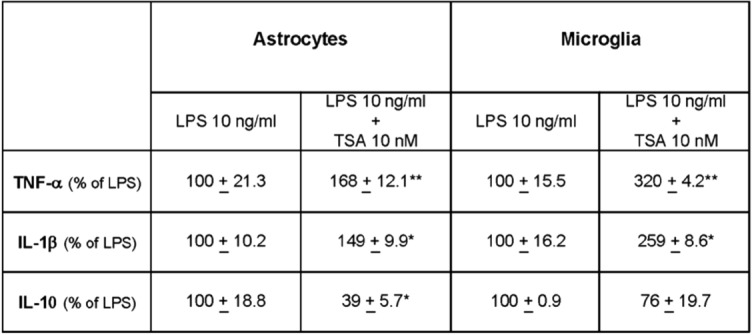
Effect of prolonged exposure to 10 nM TSA on cytokine release in LPS-stimulated microglia and astrocytes. Primary cultures of microglia and astrocytes were incubated with 10 ng/ml LPS in the absence or presence of 10 nM TSA for 24 h. The release of TNF-α, IL -1β and IL -10 was then measured. Results are expressed as a percentage of LPS. Data are the mean ± SEM of two or three independent experiments. Statistical analysis was performed by one-way ANOVA followed by Tukey’s post hoc test. Significance is indicated as * *p* < 0.05, **p < 0.01 for LPS + TSA 10 nM vs LPS.

### Influence of the pre-treatment schedule with TSA and/or LPS on cytokines release in mixed glial cells

**Fig. 2:**
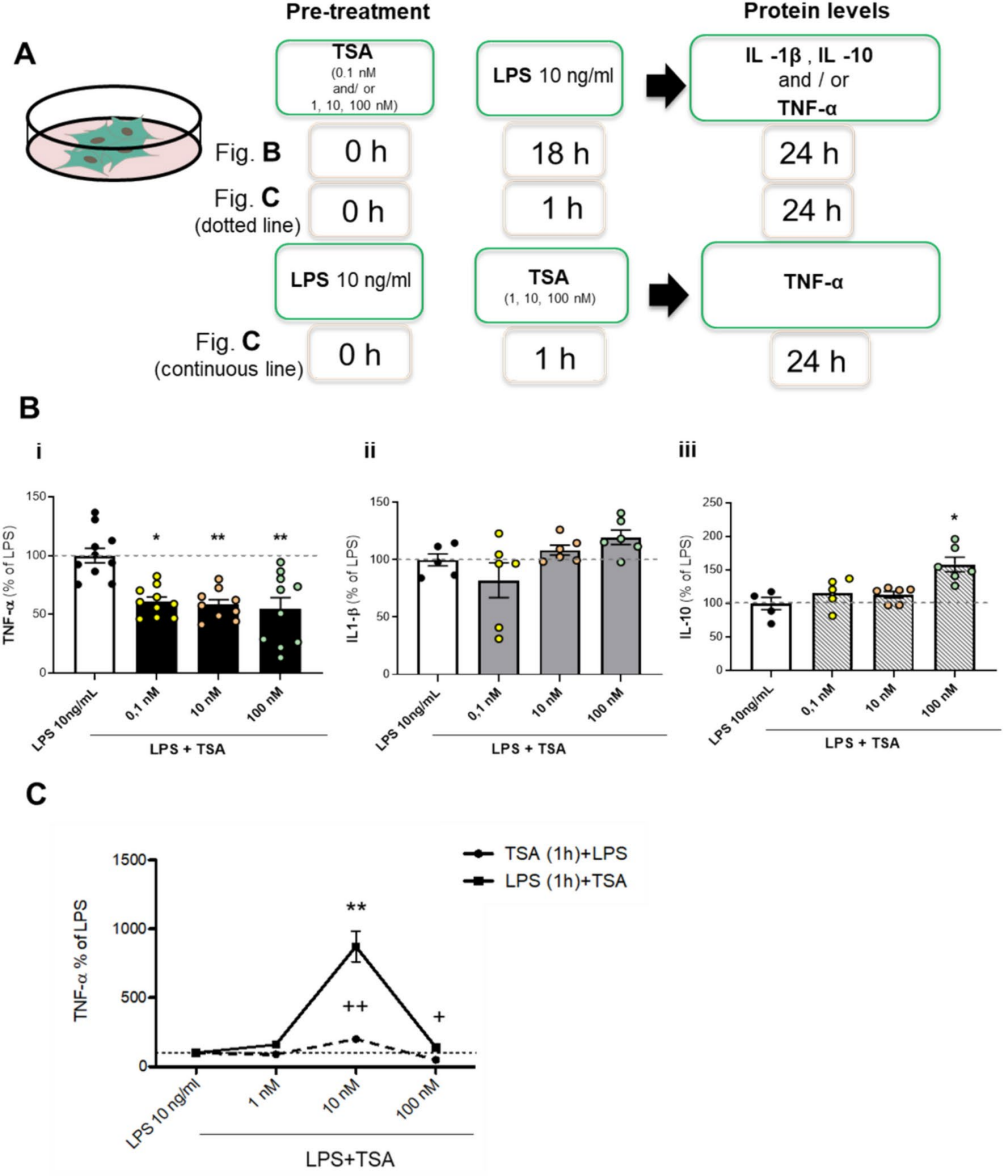
Effect of TSA or LPS pre-treatment on cytokines release in glial cells. (**A**) Experimental design. TSA pre- treatment: glial cells were treated with different concentrations of TSA (0.1 and/or 1, 10, 100 nM). LPS (10 ng/ml) was added after 18 h (for TSA long-term exposure), (panel B, refer to (B)); or 1 h (for TSA short -term exposure), (panel C, refer to (C) with dotted line). LPS pre- treatment: glia cells were treated with LPS (10 ng/ml) followed by TSA (1, 10, 100 nM) after 1 h (panel C, refer to (C) with continuous line). (**B**) Protein levels of TNF-α (i), IL -1β (ii) and IL -10 (iii) were measured in the culture medium after 24 h by biological assay or ELISA for the condition described in (A), panel B. (**C**) TNF -α release after short pre-treatment (1 h) with TSA (1, 10, 100 nM) or LPS (10 ng/ml), according to panel C of (A). Data are mean ± SEM of two to four independent experiments in triplicate. Statistical analysis was performed by one-way ANOVA followed by Tukey’s post hoc test, *p < 0.05, **p < 0.01, LPS + TSA vs LPS; + *p* < 0.05, +  + *p* < 0.01, TSA + LPS vs LPS.

Primary cultures of glial cells were exposed to TSA (0.1–10 - 100 nM) for 18 h (Fig. 2A—panel B and Fig. 2B), followed by treatment with 10 ng/ml LPS. TNF-α, IL-1β and IL-10 release were evaluated after 24 h (Fig. 2B). By using this paradigm, the production of TNF-α at all tested concentrations was significantly reduced (Fig. 2B - inset i). On the other hand, IL-1β and IL-10 were not significantly affected, except for IL-10 at the highest dose of TSA, 100 nM, where an increase in the level of this cytokine was observed (Fig. 2B - inset ii and iii, respectively).

Primary cultures of glial cells were exposed to two additional conditions and TNF-α was measured as representative cytokine (Fig. 2A - panel C and Fig. 2 C).

Condition (a): glial cells were treated with TSA at concentrations of 1,10 and 100 nM for 1 h, followed by exposure to 10 ng/ml LPS for 24 h (Fig. 2A - panel C; Fig. 2C - dotted line). The results indicated that short pre-treatment with 10 nM TSA significantly increased TNF-α production, whereas the highest dose of TSA (100 nM) significantly reduced TNF-α levels.

Condition (b): glial cells were exposed to LPS for 1 h followed by treatment with TSA at concentrations of 1, 10 and 100 nM for 24 h (Fig. 2A - panel C; Fig. 2C - continuous line). The pre -treatment with LPS followed by the addition of TSA 10 nM significantly increased TNF-α production.

### Dose-dependent effects of TSA on the expression of pro- and anti-inflammatory cytokines in LPS-co-stimulated primary alveolar macrophages

**Fig. 3:**
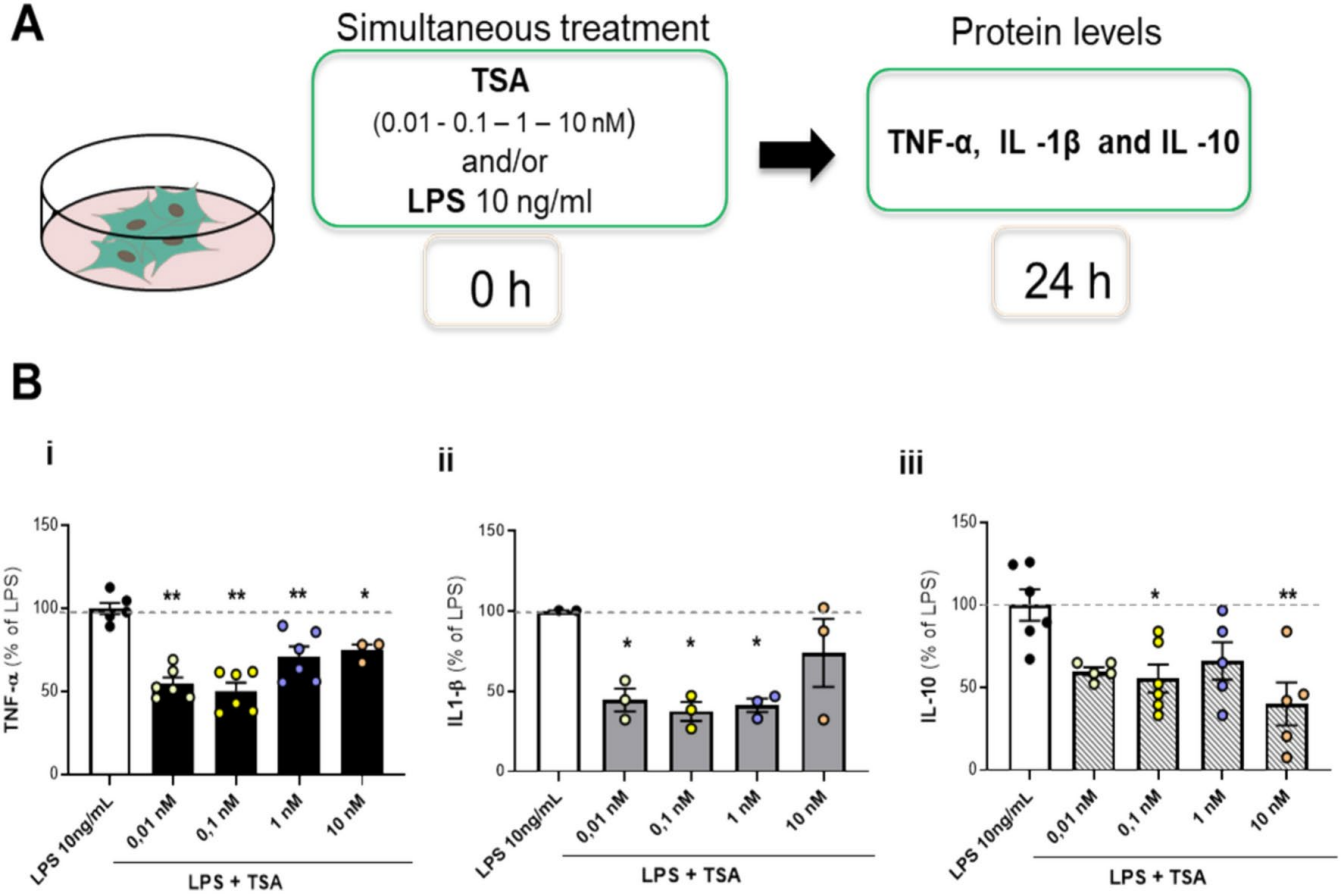
Effect of prolonged exposure to TSA on cytokines release in LPS-stimulated macrophages. (**A**) Experimental design for prolonged TSA exposure. (**B**) Primary cultures of alveolar macrophages were treated with 10 ng/ml LPS in the absence or presence of increasing concentrations of TSA (0.01, 0.1, 1, 10 nM) for 24 h. Levels of TNF -α (i), IL -1β (ii) and IL-10 (iii) were measured by biological assay or ELISA. The results are expressed as a percentage of LPS. Data are mean ± SEM of two independent experiments in triplicate. Statistical analysis was performed with one-way ANOVA followed by Tukey’s post hoc test, *p < 0.05, **p < 0.01 for LPS + TSA vs LPS.

Primary cultures of alveolar macrophages were treated with 10 ng/ml LPS in the absence or the presence of increasing concentrations of TSA (0.01, 0.1, 1, 10 nM) for 24 h (Fig. 3A). LPS did not affect macrophage viability (cell viability: control = 100% ± 2.2, LPS 10 ng/ml = 94.7% ± 12.1), nor did any TSA concentrations tested, with or without LPS 10 ng/ml (cell viability relative to the highest concentration: control = 100% ± 3.6, TSA 10 nM = 81.50 ± 10.5, LPS 10 ng/ml + TSA 10 nM = 94.1% ± 1.20). In contrast to what was observed in glial cells, co-exposure of primary alveolar macrophages to increasing concentrations of TSA and LPS resulted in reduced production of both pro-inflammatory (TNF-α, IL-1β) and anti-inflammatory (IL-10) cytokines compared to alveolar macrophages treated solely with LPS (Fig. 3B).

### Dose-dependent effects of SAHA on the release and the expression of cytokines in LPS-stimulated glial cells

**Fig. 4:**
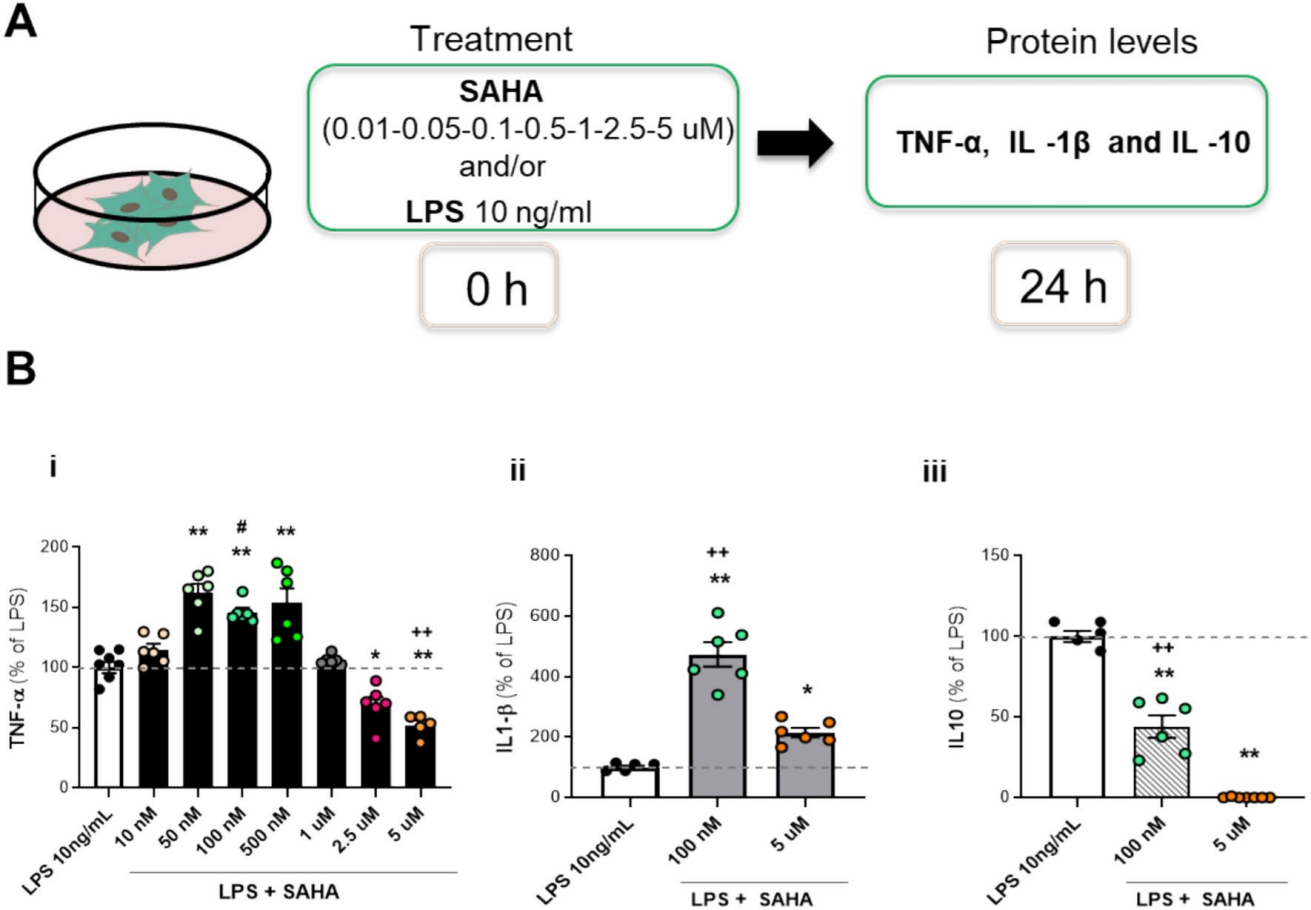
Effects of prolonged exposure to SAHA on cytokine release in LPS-stimulated glial cells. (**A**) Experimental design. Glial cells were treated with 10 ng/ml LPS in absence or presence of different concentrations of SAHA for 24 h. (**B**) TNF—α (SAHA: 50, 100, 500 nM, 1, 2.5, 5 mM) (i), IL—1 β (SAHA: 100 nM and 5 mM) (ii) and IL—10 (SAHA: 100 nM and 5 mM) (iii) release was measured in the culture medium by biological assay or ELISA. The results are expressed as a percentage of LPS. Data are mean ± SEM of two independent experiments in triplicate. Statistical analysis was performed with one-way ANOVA followed by Tukey’s test, *p < 0.05, **p < 0.01 LPS + SAHA *vs* LPS; +  + *p* < 0.01, LPS + SAHA 100 nM vs. 5 uM; # *p* < 0.05 LPS + SAHA 100 nm vs 1uM, 2,5 uM, 5uM. Although not shown in the graph, the following significant differences were also observed: TNF-α (i): Comparisons of LPS + SAHA 10 nm vs 50 nM, 100 nM, 500 nM, 2,5 uM, 5uM (p < 0.01); Comparisons of LPS + SAHA 50 nm vs 1uM, 2,5 uM, 5uM (p < 0.01); Comparisons of LPS + SAHA 500 nM vs 1uM, 2,5 uM, 5uM (p < 0.01); Comparisons of LPS + SAHA 1uM vs 2,5 uM, 5uM (p < 0.01).

The effects of the alternative HDAC inhibitor SAHA, were subsequently investigated. Primary cultures of mixed glial cells were treated with 10 ng/ml LPS either alone or with increasing concentrations of SAHA (ranging from 10 nM to 5 μM) for 24 h (Fig. 4A). None of the SAHA concentrations tested, weather in the presence or absence of LPS, affected glial cell viability (data not shown). The results indicate that nanomolar concentrations of SAHA up to 500 nM significantly enhanced LPS-induced TNF-α release, while micromolar concentrations of SAHA caused a dose-dependent reduction in TNF-α levels (Fig. 4B, inset i). Based on these observations, 100 nM and 5 μM SAHA were selected to investigate the co-treatment effects with 10 ng/ml LPS for 24 h on IL-1β and IL-10 release. Both SAHA concentrations enhanced IL-1β release, with the effect being significantly more pronounced at 100 and diminishing at 5 μM (Fig. 4B, inset ii). Furthermore, SAHA dose-dependently decreased LPS-stimulated IL-10 releases in glial cells (Fig. 4B, inset iii).

### Dose-dependent effects of selective class I or II HDAC inhibitors on the expression of pro- and anti-inflammatory cytokines in LPS-co-stimulated mixed glial cells

**Fig. 5:**
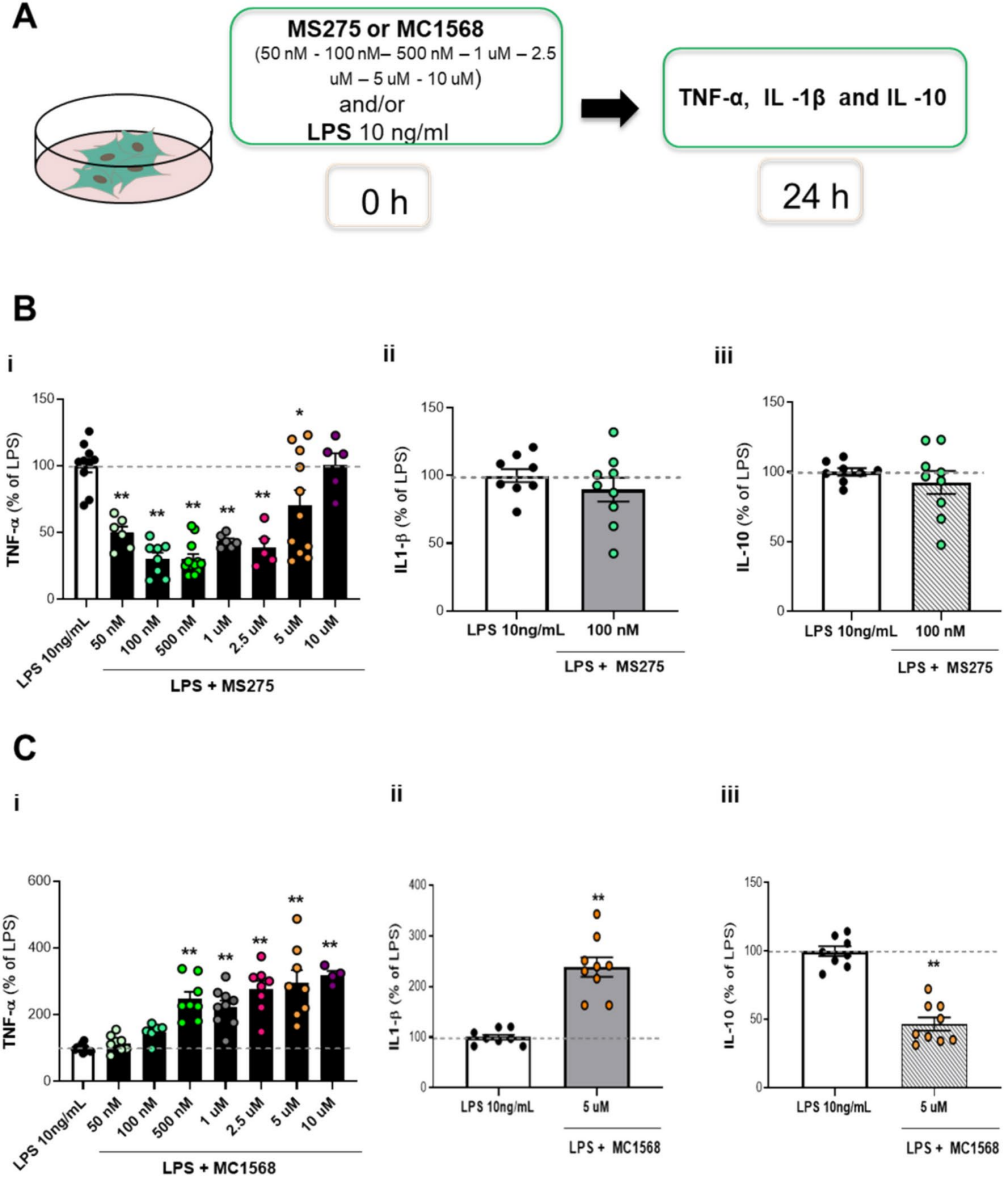
Effect of selective class I HDACs (MS275) and class II HDACs (MC1568) on cytokines release in LPS-stimulated glial cells. (**A**) Glial cells treated with 10 ng/ml LPS alone or with MS275 (**B**) or MC1568 (**C**) for 24 h. TNF -α (MS275 and MC1568: 50, 100, 500 nM, 1, 2.5, 5, 10 mM) (i), IL -1β (MS275: 100 nM; MC1568: 5 mM) (ii) and IL—10 (MS275: 100 nM; MS275: 5 mM) (iii) release was measured in the culture medium by biological assay or ELISA, panel (**B**) and (**C**). The results are expressed as a percentage of LPS. Data are mean ± SEM from two to four independent experiments in triplicate. Statistical analysis was performed with one-way ANOVA followed by Tukey’s test, *p < 0.05, **p < 0.01 LPS + MS275 or MC1568 *vs* LPS. Although not shown in the graph, the following significant differences were also observed: (**B**) TNF-α (i): Comparisons of LPS + MS275, 50 nM, 100 nM,500 nM,1uM, 2,5 uM vs 10uM (p < 0.01); Comparisons of LPS + MS275, 100 nM, 500 nM vs 5uM (p < 0.01). (**C**) TNF-α (i): Comparisons of LPS + MC1568 100 nM vs 500 nM, 2,5 uM, 5 uM, 10 uM (p < 0.05); Comparisons of LPS + MS275, 50 nM vs 500 nM, 1uM,2,5 uM, 5 uM, 10 uM (p < 0.01).

After evaluating the effects of non-selective HDAC inhibitors on cytokine release, we investigated class-selective inhibitors. Using the same conditions, glial cells were exposed to 10 ng/ml LPS with or without increasing concentrations of MS275 (a class I HDACi) or MC1568 (a class II HDACi) for 24 h (Fig. 5A). MS275 significantly reduced TNF-α release in a dose-dependent manner but lost the effect at 10 μM in mixed glia cells co-treated with LPS (Fig. 5B, inset i). Micromolar concentrations of MC1568 enhanced TNF-α release (Fig. 5C, inset i). We selected the doses of 100 nM for MS275 or 5 μM for MC1568, as representative concentrations to treat primary glial cultures with or without 10 ng/ml LPS. IL-1β and IL10 levels were measured 24 h post-exposure. As shown in Fig. 5B, C (inset ii and iii), 100 nM MS275 did not affect IL-1 β and IL10 levels (Fig. 5B, inset ii and iii). However, 5 μM MC1568 increased the release of IL-1β and significantly reduced the production of IL-10 in LPS-stimulated glial cells (Fig. 5C, inset ii and iii).

### Microarray analysis of genes differentially regulated by 100 nM and 5 μM of SAHA

#### Gene expression modulation after co-exposure of glial cells to LPS and SAHA

**Fig. 6:**
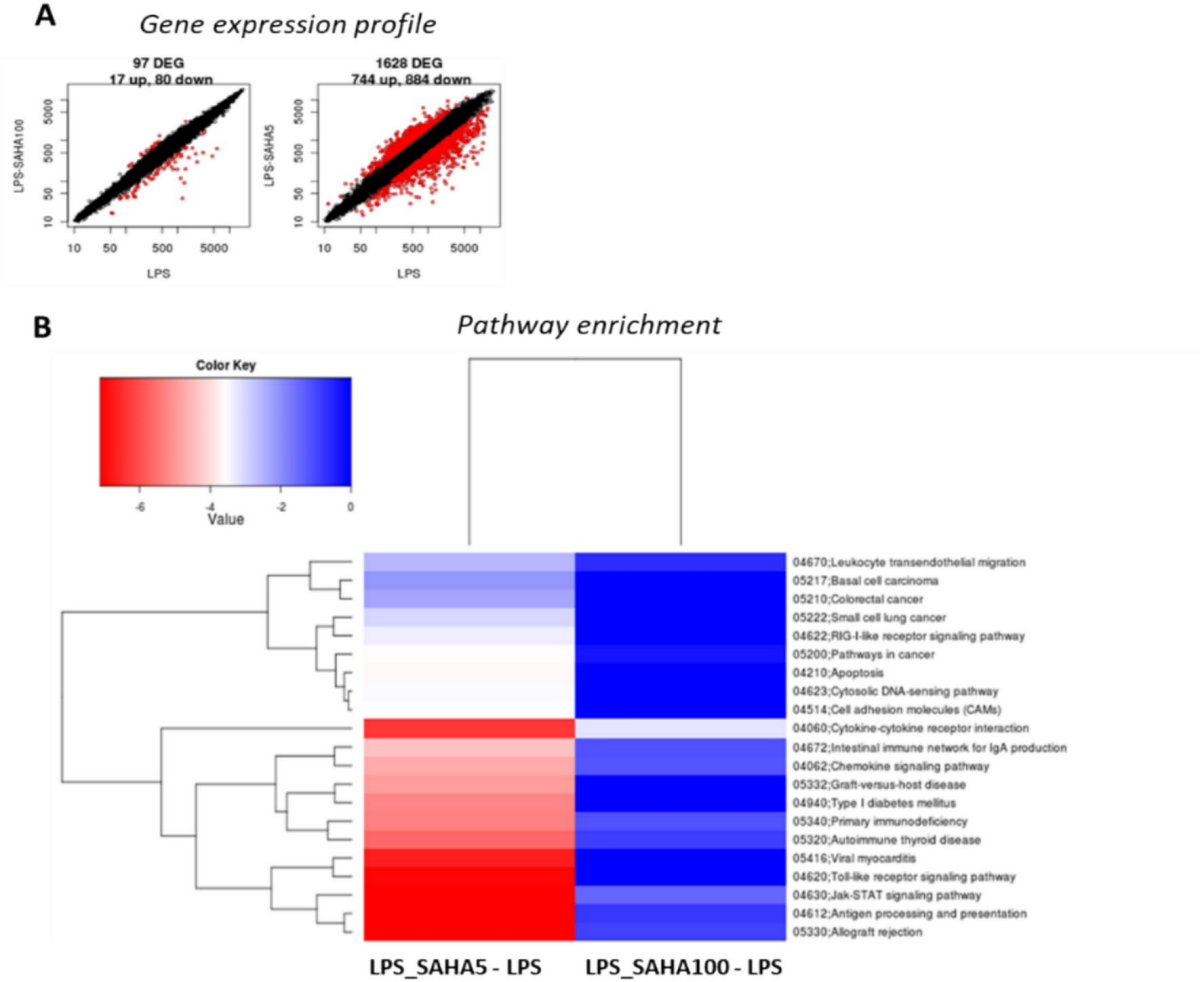
(**A**) Scatter plots of log2 intensity values of DEGS. Total number of DEGs (red dots) identified for glial cells treated with SAHA 100 nM or SAHA 5 uM compared to LPS (10 ng/ml) condition. DEGs identified with the Limma algorithm with a threshold value of 0.01. On the y-axis are reported the means of the replicates of the treatment conditions and on the x-axis the mean across the replicates of the reference condition (LPS 10 ng/ml). (**B**) Pathway enrichment analysis. KEGG functional summary reporting the enrichment p-values less than 0.05 (log10 of p-value) for the different annotation terms LPS_SAHA100 - LPS and LPS_SAHA5—LPS. The color scheme is based on a simple scale (reported above) painting pathways (blu down – and red up- regulated) by automatic analysis using basic analysis.

To elucidate SAHA’s dual effect, we performed a comprehensive transcriptomic analysis in glial cells using microarray technology. Gene expression was evaluated following co-exposure to 10 ng/ml LPS and either 100 nM or 5 μM SAHA, or LPS alone, for 24 h. Transcription profiles were compared between LPS-treated cells with LPS and/or 100 nM SAHA (LPS_SAHA100 – LPS), or LPS and/or 5 μM SAHA (LPS_SAHA5 – LPS), as illustrated in Fig. 6A, B. A total of 97 differentially expressed genes (DEGs) were identified in the LPS_SAHA100 with 17 upregulated and 80 genes downregulated. In contrast, the LPS_SAHA5 group exhibited a far more substantial transcription impact, with 1628 DEGs, including 744 upregulated and 884 downregulated genes (Fig. 6 A, Table S1).

Notably, only a small subset of DEGs were shared between the two SAHA concentrations (Table S1). Among these shared genes, those involved in immune response modulation (i.e., *Fap, Gcg, Ccl22, Slamf1, Cxcl13*) were strongly repressed, while some signal transduction pathways were increased (i.e., *Plekhh3*) with both concentrations.

For the comparison LPS_SAHA100 – LPS, several genes were uniquely downregulated, including *Gpr183*, *Rgs2*, and *Rgs1*, which are associated with G-protein coupled receptors signaling. For LPS_SAHA5 – LPS comparison, downregulated genes, including *Clec4e*, *Has2*, *Stat1*, *Jak2* and *Ccr5*, play roles in inflammatory and immune signaling. Conversely, unique upregulated genes in the LPS_SAHA100 – LPS comparison included *LOC302473*, *Zfp57*, *Rreb1*, and *Zbtb7b*, all involved in transcriptional regulation of immune response. For LPS_SAHA5 – LPS comparison, upregulated genes such as *Gdf15* and *Plag1* were linked to cytokine and transcription factor activity (Table S1).

Gene expression profiles were then compared through analysis of canonical pathways enrichment, KEGG, as described in Materials and Methods. Enrichments significantly modulated are reported in Fig. 6B, according to DEG functional annotation analysis.

Most of the pathways were modulated based on the dose used, with the higher SAHA concentration (LPS_SAHA5) having a stronger overall impact. Among significantly altered pathways “Cytokine-cytokine receptor interaction” and “JAK-STAT signaling pathway” were identified in both comparisons, with DEGs within these pathways further explored for potential opposing transcript regulations based on dose (Table 2). Few DEGs were commonly altered across both doses, while certain genes were specifically regulated in the LPS_SAHA5—LPS comparison. Notably, IL-10 was significantly downregulated with both doses and in all pathways analyzed, consistent with earlier observations in glial cells.

**Table 2:**
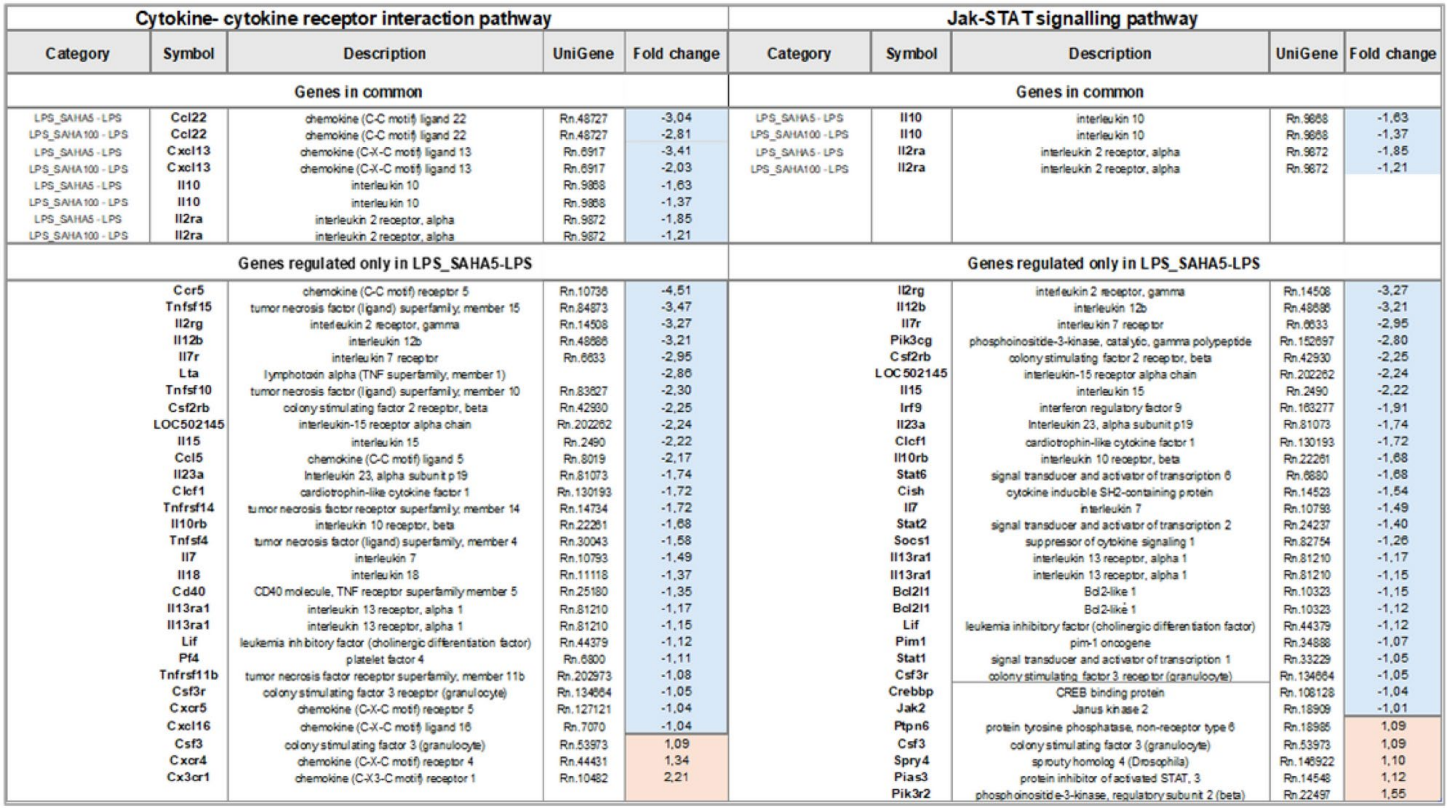
DEGs members were detected as significantly expressed for the condition LPS_SAHA5—LPS and LPS_SAHA100 – LPS for the common pathways regulated, “cytokine—cytokine receptor interaction” and “JAK/STAT signalling pathway”. Color schema follows the statistic of differentially expressed (blue stands for down – and red for up-regulated genes, NA- not detected).

Gene ontology (GO) enrichment analysis (Fig. S2) further highlighted dose-specific differences, with pathways related to chemokine activity, cytokine receptor activity, and immune system regulation, with the higher dose of SAHA.

#### Modulation of gene expression by SAHA in absence of LPS

To evaluate the effects of SAHA treatments on glial cells in the absence of prior LPS-induced inflammatory stimulation, we analyzed the transcription profiles of the SAHA100 - CTRL and SAHA5 - CTRL groups (Fig. S3 and S4).

For SAHA100 - CTRL comparison, a total of 18 DEGs were identified, with two genes upregulated and 16 downregulated. In contrast, treatment with 5 uM SAHA resulted in 849 genes differentially regulated, including 339 upregulated and 510 downregulated genes (Fig S3A). The overlap between the two experimental conditions revealed only a small number of shared DEGs (Table S3). Among these, genes involved in inflammatory response such as Neuropeptide Y (*Npy*), Interferon-induced protein 1 (*Ifit1*), Ermin (*Ermn*) and GNAS (*Gnas*) exhibited dose-dependent downregulation in response to SAHA. KEGG pathway analysis, alongside assessments of biological processes (BP), molecular functions (MF), and cellular components (CC), showed significant enrichment in inflammatory and immune-related processes (Fig. S3 and S4, respectively).

#### Modulation of gene expression for mixed glial cells with or without LPS

To investigate treatment-induced differences between control (SAHA-CTRL) and LPS-stimulated (SAHA-LPS) cells, we analyzed DEGS and visualised the overlap using a Venn diagram (Fig. S5). The diagram highlights two sets of comparisons: (a) *SAHA 100* - *CTRL* and *SAHA 100* - *LPS* and (b) *SAHA 5* - *CTRL and SAHA 5* - *LPS.* The intersections provide insight into the number of genes potentially involved in shared functions.

For condition a) with SAHA 100 nM, 14 genes were modulated for the control condition, 93 genes were altered for SAHA 100- LPS -stimulated cells, and only 4 genes were shared between these two groups. For condition b) with SAHA 5 μM, 335 genes were modulated in the absence of LPS, 1114 genes were altered in the presence of LPS, and 514 overlapped between the two conditions (Fig. S5).

DEGs significantly modulated in the overlapping regions are listed in Table S3. In condition (a), *Npy* (neuropeptide Y) was consistently downregulated, while the remaining three genes could not be identified (NA). In condition (b), genes with lower fold-change values included *Npy*, *Nav3* (neuron navigator 3), and *Fst* (follistatin). Conversely, *Napepld* (N-acyl phosphatidylethanolamine phospholipase D) and *Plag1* (Pleiomorphic adenoma gene 1) displayed the highest positive fold-change values. These genes are known to regulate neuroinflammation by modulating cellular homeostasis and signal transduction. Notably, *Npy* was consistently reduced under both SAHA treatment concentrations.

## Discussion

The role of HDAC activity in regulating neuroinflammation has received considerable attention within the context of neurodegenerative diseases^21^; however, previous studies have yielded conflicting results, particularly due to varying findings from different cell types and experimental paradigms^11,35,36^. This study aimed to address these inconsistencies by examining how different HDACi influence inflammatory responses in glial cells, with an emphasis on dose-dependent effects of both pan and specific HDACi. By doing so, it elucidates specific outcomes affected by HDACi in glial cells, offering insights that could inform future research in this field.

The results presented highlight the complex, context-specific actions of HDACi in modulating inflammatory responses. The findings underscore the importance of considering multiple variables, such as HDACi concentrations, timing of exposure relative to the inflammatory stimulus, and the spectrum of inhibition, to fully understand the therapeutic potential and limitations of these compounds.

TSA and SAHA exert dose-dependent modulation of LPS-induced cytokines release in glial cells, with significant differences observed between their effects on pro-inflammatory (TNF-α, IL-1β) and anti-inflammatory (IL-10) cytokines. TSA (up to 10 nM) enhances LPS-induced TNF-α and IL-1β release while simultaneously reducing IL-10 levels. These effects are apparent following a 24 h co-exposure of glial cells to TSA and LPS and are reproducible in both primary astrocytes and microglia. In contrast, long-term (18 h) pre-treatment with TSA does not affect IL-1β, increases LPS-induced IL-10 at the highest concentration of 100 nM, and reduces TNF-α at all tested concentrations. A similar reduction in TNF-α is observed after a short-term (1 h) pre-treatment but only at the highest concentration of 100 nM. Additionally, a potentiation of TNF-α is observed when LPS is administered 1 h prior to TSA administration.

These findings align with various studies where co-exposure of TSA (nM) and LPS for 24 h potentiates IL-6 release in B9 microglial and hippocampal slice cultures^34^, while a reduction of production and release of different pro-inflammatory and anti-inflammatory cytokines is evident upon pre-treatment with TSA followed by LPS exposure in primary microglial cells^37^, BV2 microglia^38^ or in co-exposure for periods shorter than 12 h^34^. Our results, therefore, underscore the critical importance of the timing of TSA administration relative to the inflammatory stimulus in determining the overall effect on inflammation in glial cells, thereby providing clarity to the seemingly contradictory data present in the literature.

In addition to timing, TSA can modulate the inflammatory response differently based on the cell type. Primary alveolar macrophages exhibited a distinct response profile. A 24 h co-exposure to LPS and TSA resulted in a dose-dependent reduction of both pro-inflammatory cytokines (TNF-α and IL-1β) and the anti-inflammatory cytokine IL-10 compared to macrophages treated solely with LPS. A similar response has been reported in RAW264.7 macrophages^39^.

SAHA exhibits a dual role, manifesting both pro- and anti-inflammatory effects depending on the concentration. At nanomolar concentrations, SAHA enhances LPS-induced TNF-α and IL-1β; however, at micromolar concentrations, it reduces the levels of both cytokines and decreases IL-10 in a dose dependent manner. It was shown that the concomitant administration of micromolar concentrations of SAHA and LPS led to a reduction in various LPS-induced cytokines, irrespective of the cell type. This observation suggests a “universal” anti-inflammatory effect at these concentrations. This effect has been observed in primary glial cultures^11^, microglia^37^, BV2 microglia^40^, cutaneous T-cell lymphoma cells^41^, RAW264.7 macrophages^39^, bone marrow cells^29^. To our knowledge, no data regarding the effect of SAHA at nM concentration is reported in the literature.

Selective HDACi are crucial to understand the molecular mechanism of specific HDAC isoforms and the distinct and overlapping effects of class I and II HDAC inhibition on inflammation. The use of selective inhibitors of class I (MS275) and class II (MC1568) under identical treatment conditions (co-exposure for 24 h) and within the same nM to microM range as SAHA, demonstrated dose-dependent effect across the concentration range while showing distinct differences between the two substances. Specifically, MS275 reduces TNF-α, while MC1568 increases it in a dose-dependent way. The modulation of IL-1β and IL-10 also differs between these inhibitors; MS275 does not affect the production of either cytokines, whereas MC1568 increases IL-1β and decreases IL-10 in a dose-dependent manner. Several studies have shown that class I HDACi MS275 reduces neuroinflammation in a mouse model of Alzheimer’s disease^42–44^ and suppresses cytokine expression in murine microglia activated with LPS^40^. Although there is substantial evidence of MC1568’s ability to modulate inflammation depending on the cell types^20,45,46^, to our knowledge, apart from our data, only few observations were reported supporting MC1568 capacity to influence the glial inflammatory response in in vivo rat model of Parkinson’s disease^47^.

Based on recent data, it remains challenging to elucidate whether the dual effect of SAHA is linked to differential affinities for Class I and Class II HDACs. SAHA shares overlapping pKi values between Class I HDAC (1, 2, 3,8) ranging from 6.3 to 8.9, Class IIa (5 and 9) ranging from 5.4 to 7.2, and Class IIB (6,10) ranging from 7.3 to 8.8^48^.

For the foregoing, our results underscore the importance of the dosage in the dualistic effects of pan-HDACi on the inflammatory response. In particular, our findings indicate that TNF-α is consistently modulated by all examined HDACi, suggesting that TNF-α could serve as a reliable biomarker for assessing the impact of HDAC inhibition on inflammatory processes, regardless of the cell type.

Our study focused exclusively on LPS as the stimulating trigger, which may limit the broader applicability of our findings to various inflammatory contexts. Nevertheless, HDACi both reduce and potentiate cytokine production observed with different triggers, such as Pam3CSK (a TLR2 activator)^34^, Flagellin (TLR 5 activator)^34,49^, ODN1826 (a TLR 9 activator)^34^, IL1b (IL1R activation)^50^ and cells from peripheral blood of patients with Seazary syndrome^51^, thus already prompted to produce cytokines^41^. This suggests that the modulation of cytokines production by HDACi is not contingent upon the activation of specific receptors.

In our experimental paradigm, the choice of LPS allowed for a more detailed examination of the specific effects of HDACi on glial cells in a controlled environment. The LPS model is well-established in neuroinflammation research and extensively used^52^, providing a clear framework for investigating the mechanisms by which HDACi modulate inflammatory responses and facilitating comparisons between different studies. To enhance the robustness of our conclusions and address real life exposure and potential limitations, it is necessary to investigate a wider array of inflammatory stimuli, including cytokines, neurotoxins, and other pathogen-associated molecular patterns (PAMPs). This data would provide a more comprehensive understanding of the therapeutic potential of HDACi in diverse inflammatory conditions.

HDACi may act on both histone and non-histone proteins, potentially regulating processes beyond transcription. In our experimental conditions, TSA modulation of mRNA expression mirrors the effect on protein production for all three cytokines, suggesting the involvement of transcriptional mechanisms.

Our transcriptomic analysis identified patterns of gene expression at nanomolar (100 nM) and micromolar (5 µM) concentrations of SAHA. We observed that HDACi affects the regulation of transcripts involved in homeostatic pathways, with 5 µM SAHA being the most effective dose. SAHA was selected for gene expression analysis due to its established efficacy as a broad-spectrum HDAC inhibitor in various cell types^53^ and its clinical relevance and potential application in neurodegenerative diseases^10,21^, as previously reported in the introduction session. Unlike TSA, which has potent HDAC inhibition at very low concentrations, SAHA’s efficacy across a broader concentration range enabled us to explore both low and high concentrations in relation to inflammation and homeostatic gene regulation.

In our transcriptomic analysis, the co-stimulation paradigm (SAHA with and without LPS) was designed to assess both baseline effects and inflammation-specific modulation by HDAC inhibition. This approach enabled differentiation between SAHA’s inherent effects on glial cells and those induced under inflammatory conditions, which is crucial for understanding how SAHA might function in pathological versus homeostatic states.

Using LPS to simulate an inflammatory response, we evaluated SAHA’s ability to modulate gene expressions and pathways associated with cytokine signaling and inflammatory cytokine release.

These array data served to confirm and validate some of our molecular findings. Indeed, our transcriptome analysis revealed that both concentrations of SAHA reduced IL-10 expression. Although TNF-α and IL-1β expression remained unchanged, we observed alterations in key transcripts involved in their downstream signaling. This suggests that SAHA may indirectly modulate cytokine maturation by influencing critical regulatory factors within these pathways. Specifically, in the comparison LPS_SAHA5 *vs.* LPS, several members of the tumor necrosis factor receptor superfamily were downregulated, while genes like *Lta*, *Casp-8* (caspase-8), *Traf4*, and *Fadd* were upregulated. These genes are involved in inflammation and cell death pathways^54^. TNF-α is known to promote apoptosis in astrocytes via the RIP1/FADD/Caspase-8 axis^55^. Additionally, CASP1, a protein that converts pro-IL-1β to active IL-1β^56^, was downregulated for 5 µM SAHA, suggesting a potential mechanism for its anti-inflammatory effects.

For the LPS_SAHA5—LPS comparison, we also noted a decrease in the expression of the IL-10 receptor beta (*Il10rb*) and an upregulation of *PIAS3*, a protein that inhibits STAT3 activation. These genes are integral to the IL-10 pathway, where IL-10 activates STAT3 to mediate anti-inflammatory responses, and PIAS3 inhibits this activation^57,58^. In contrast, at a dose of 100 nM, only IL-10 and IL-2Ra (an IL-1β receptor antagonist) were downregulated.

Based on the analysis of KEGGs pathways, SAHA at 5 µM and 100 nM primarily cause transcriptional dysregulation of JAK signalling and altered activation of cytokines cascade signalling. Indeed, only two signalling pathways were differentially modulated by both doses of SAHA: “*JAK/STAT* pathway” and “Cytokines-cytokines receptor interaction pathways”.

The JAK-STAT pathway is a well-established signaling system that plays a crucial role in various disease states, particularly neuroinflammatory conditions^59^. In this study, we found that 5 µM SAHA modulated key genes within this pathway, including the downregulation of *STAT1, STAT2, STAT6, JAK2,* interleukins, interferons, and *BCL2L1*, alongside the upregulation of negative feedback regulators such as *PIAS, Sprouty,* and *P13K*. These findings are consistent with previous research^60–62^, supporting HDACi^63^, as well as, SAHA’s anti-inflammatory effects via JAK-STAT pathway attenuation in different conditions^19,64,65^.

Although our results align with existing literature, a limitation is the lack of external validation for our microarray analysis. While the findings provide insights into HDACi-modulated gene expression and related pathways in glial cells, further validation would strengthen their robustness. Our approach identified broad gene networks and pathways affected by SAHA; however, the biological significance of the individual genes requires more focused functional studies for validation.

In conclusion, this study addresses discrepancies in the existing literature regarding the effects of HDACi on inflammation in glial cells. It revealed a variable response to HDACi depending on cell type, HDACi dosage, protocol and HDACi specificity. By comparing broad-spectrum and class-selective HDACi and employing transcriptomic analysis, the study sheds light on the dualistic regulation of inflammation. One strength of this study is its ability to pinpoint dose-dependent responses of HDACi, which is essential for understanding their dual effects on inflammation – a critical factor when considering therapeutic applications. By using global gene expression analysis, we mapped potential molecular pathways modulated by SAHA. This approach offers a framework for identifying and validating potential therapeutic targets, which may be crucial for further investigation. While our findings provide insight into how HDAC inhibition can influence inflammation in glial cells, the study’s applicability to complex in vivo contexts remains uncertain, as in vivo environments involve more interacting cell types and signaling pathways. Moreover, factors such as blood–brain barrier permeability, compound metabolism, and interactions with other systemic processes make it challenging to predict how these HDAC inhibitors will act in a whole-organism setting. Future research should aim to validate the biological significance of identified genes through targeted functional studies and establish optimal HDACi dosages and protocols for effective therapeutic application in neuroinflammation and neurodegenerative disease contexts.

Our study suggests that HDAC inhibition holds promise for neuroprotective therapies, but further validation and exploration of different experimental conditions are warranted to enhance the applicability of these findings in clinical practice.

## Materials and methods

### Reagents

All cell reagents, unless otherwise specified, were purchased from Sigma-Aldrich Co. (St. Louis, MO, USA) at the highest purity available. Lipopolysaccharide (LPS) from *Escherichia coli* serotype 0127: B8 was obtained from Sigma-Aldrich Co. (L4516) and the stock solution was dissolved in PBS to a final concentration of 5 mg/ml. Tricostatin A was purchased from Sigma-Aldrich Co. (T8552), Suberoylanilide hydroxamic acid was purchased from Cayman (Michigan, USA) (10009929), MS275 and MC1568 were synthetized and were kind gifts by Antonello Mai (“Sapienza” Università di Roma). The stock solutions of inhibitors were dissolved in DMSO to a final concentration of 20 mM (final concentration of DMSO in culture medium < 0.1%). Reagents and primers (18 s: Hs99999901_s1; TNF-α: Rn99999017_m1; IL-1 β: Rn00580432_m1; IL-10: Rn00563409_m1) for real time reverse transcriptase polymerase chain reaction were from Applied Biosystems (Foster City, CA, USA).

### Cell cultures

#### Primary culture of glial cells

All experimental procedures leading to the preparation of primary cell cultures were carried out in accordance with the guidelines of the European Communities Council (Directive of November 24, 1986, 86/609/EEC), the local guidelines and regulations (Italian Legislative Decree n. 116/1992) for the care and use of laboratory animals and were approved by the Italian Ministry of Health (as indicated in Digs n. 295/2012-A). The University of Milan follows the Arrive guidelines.

Sprague Dawley rats (Charles River, Calco, Italy) were kept under standard animal housing (temperature 20 ± 2 °C; humidity 60–70%) with food and water ad libitum, under a 12 h–12 h light/dark cycle (lights on from 7:00 a.m. till 7:00 p.m.). Rats were anaesthetised with isoflurane before suppression.

Primary cultures of glial cells were prepared from 2-day-old newborn rats (Sprague–Dawley, Charles River, Calco, Italy), following a standardized procedure^66,67^.

Cerebral hemispheres were freed of the meninges and were mechanically disrupted; cells were dispersed in a solution of trypsin 2.5% and 1 mg/ml DNAse, filtered through a 100-um nylon mesh. Cells were seeded in 24-well plates for the treatments of mixed glial cells (5 × 10^4^ cells per well) and in a 75 cm^2^ flask for the preparation of microglia and astrocytes (5 × 10^6^ cells per flask), in Minimum essential Eagle’s Medium (MEM) supplemented with 10% fetal calf serum (FCS), 0.6%glucose, 0.1 mg/ml streptomycin, 100 IU/ml penicillin and 2 mM L-glutammine^66,67^. Glial cultures were fed twice a week and grown up to confluence at 37 °C in a humidified incubator with 5% CO_2_. Cells were treated after 10–15 days from plating when they reached confluence.

#### Primary cultures of astrocytes and microglia cells

A layer of astrocytic cells was obtained through vigorous shaking of a confluent 10-day-old monolayer of mixed glial cells, as described by^68^. Cultures of enriched astroglia were treated further with 5 mM L-leucine methyl ester to eliminate microglia (97% homogeneity). Isolated astroglial preparations were then plated in 24-well plates (10^5^ cells per well) in MEM with supplements as described above and treated two days after seeding. Microglia were isolated by shaking glial cultures at 800 × *g* for 2 h. Microglia, which dislodged into the medium, were purified by plating for 30 min in 24-well plates (10^5^ cells per well) in MEM with supplements as above and FCS 15%. Contaminating cells were removed with supernatant. These conditions allowed us to obtain highly enriched microglial cultures with 98% homogeneity, as assessed by immunocytochemistry with *Griffonia simplicifolia* isolectin B_4._

Griffonia simplicifolia isolectin B4 (IB4) was used as a marker to selectively identify microglia due to its ability to bind specific glycosylation patterns unique to these cells in the CNS^69^. The treatments of microglia were performed the day after the plating.

#### Primary culture of alveolar macrophages

Alveolar macrophages were collected by bronchoalveolar lavage as previously described^70^. Recovery was 10–15 × 10^6^ cells per rat, of which > 98% were macrophages, as assessed by Giemsa stain. Once washed and resuspended to 10^6^ viable alveolar macrophages/ml, the cells were seeded in 24-well plates (3 × 10^5^ cells per well) and were allowed to adhere to plastic plates in RPMI 1640 containing 10% FCS, streptomycin (0.1 mg/ml), penicillin (100 International Units/ml), L-glutammine (2 mM) and gentamicin (50 ng/ml) for 1 h at 37° in 5% CO_2_, and then treated.

### Viability assay

Cell viability was measured by the 3-(4,5-dimethyl-thiazol-2-yl)-2,5-diphenyltetrazolium bromide (MTT) assay^71^. MTT tetrazolium salt was dissolved in serum-free medium to a final concentration of 0.75 mg/ml and added to the cells (glia or macrophages) after 24 h of treatment with ± 10 ng/ml LPS ± 100 nM TSA. Cells were then incubated for 3 h at 37 °C. The medium was then removed and formazan extracted with 1N HCl:isopropyl alcohol (1:24). Absorbance of the resulting solutions was read at 595 nm in a microplate reader (Molecular Devices, Sunnyvale, CA). Results are expressed as the percentage of control cells ± Standard Error of the Means (SEM).

### Sample treatments

#### Glial cell treatments

For this study, glial cells were treated under various experimental conditions:

1. LPS exposure with TSA:
2. Glial cells were exposed to 10 ng/ml LPS for 24 h, either alone or in the presence of varying concentrations of TSA (0.01, 0.1, 1, 10, 100 nM) (Fig. 1A).
3. Short-term LPS exposure with TSA:
4. Glial cells were exposed to 10 ng/ml LPS for 1 or 6 h, either alone or in simultaneous presence of 10 nM TSA (Fig. S1).
5. TSA pre-treatment with LPS challenge:
6. Glial cells were pre-treated with TSA at different concentrations (0.1, 1, 10, 100 nM) for either 18 h (long exposure) or 1 h (short exposure) before the addition of 10 ng/ml LPS. Additionally, in a separate condition, glial cells were pre-treated with 10 ng/ml LPS, followed by the addition of TSA (1, 10, 100 nM) 1 h later (Fig. 2A).
7. SAHA treatment with LPS:
8. Glial cells were exposed to 10 ng/ml LPS alone or with varying concentrations of SAHA (50, 100, 500 nM, 1, 2.5, and 5 μM) for 24 h (Fig. 4A).
9. MS275 or MC1568 with LPS exposure:
10. Glial cells were treated with 10 ng/ml LPS in the absence or presence of different concentrations of MS275 or MC1568 (50, 100, 500 nM, 1, 2.5, 5, 10 μM) during a 24 h exposure period (Fig. 5A).

#### Primary cultures of microglia and astrocytes

Primary cultures of purified microglia and astrocytes were exposed to 10 ng/ml LPS, alone or with 10 nM TSA, for 24 h. TNF-α, IL-1β, and IL-10 release were then quantified (Table 1A).

#### Primary cultures of alveolar macrophages

Primary cultures of alveolar macrophages were treated with 10 ng/ml LPS, alone or with different concentrations of TSA (0.01, 0.1, 1, 10 nM), for 24 h (Fig. 3A).

### RNA extraction and real time reverse transcriptase-polymerase chain reaction (RT-PCR)

Total RNA was isolated from glia cells after 1, 3 and 6 h of treatment with 10 ng/ml LPS and with or without 10 nM TSA, using a commercially available kit (TriReagent from Sigma, 3934) as described by the manufacturer. For the synthesis of cDNA, 2 μg of total RNA was reverse-transcribed using a high-capacity cDNA archive kit from Applied Biosystems (Foster City, CA, USA; 4368813) following the supplier’s instructions. TNF-α, IL-1β and IL-10 gene expression were evaluated by real time reverse transcription polymerase chain reaction (Real Time RT-PCR). For PCR-analysis, Taq-Man^™^-PCR technology was used. For each PCR reaction, 40 ng of total RNA were used. The 18S ribosomal RNA was used as an endogenous reference and the quantification of the transcripts was performed by the ΔΔC_T_ method.

### Biological assay for TNF-α

TNF-α content in the medium obtained from treated cells was assayed by determining the cytotoxicity of TNF-α against sensitive L929 cells (mouse fibroblast)^70^. Briefly, L929 cells were seeded into 96-well microtiter plates (2.5 × 10^4^ cells per well) in RPMI-1460 culture medium supplemented with 10% FCS, streptomycin (0.1 mg/ml), penicillin (100 International Units/ml), l-glutammine (2 mM) and incubated for 24 h at 37 °C in a humidified atmosphere of 5% CO_2_. After removal of the medium, actinomycin D (0.6 μg/μl) was added to each well for 1 h at 37 °C. Recombinant murine TNF-α and test samples were added, and plates were incubated at 37 °C for 18 h. Cells were stained and fixed with 0.2% crystal violet in 2% ethanol for 10 min at room temperature and then lysed with 1% sodium dodecyl sulfate. Absorbance at 505 nm was detected with a microplate reader (Molecular Devices, Sunnyvale, CA). TNF-α concentration was calculated from a standard curve based on known amounts of recombinant murine TNF-α. Data were normalized to LPS and presented as percentage change in TNF-α levels ± SEM.

### IL-1β and IL-10 assay

IL-1β and IL-10 amounts were measured in the cultured media of treated cells by commercially available ELISA kit as described by manufacture (Quantikine, R&D Systems, Abingdon, UK, PRLB200 and PR1000). Data were normalized to LPS and presented as percentage change in cytokines release ± SEM.

### Microarray data analysis

Total RNA was isolated from the glia cells using the RNeasy Kit (Qiagen, Milano, Italy; 74,104) following the manufacturer’s instructions for cells treated with 100 nM or 5 μM SAHA in the absence or presence of 10 ng/ml LPS after 4 h.

RNA quality was established using Bioanalyzer (Agilent). Synthesis of cDNA was carried out starting from 5 µg of total RNA.

Microarray analysis was performed at Genopolis Consortium of Functional Genomic, Università di Milano – Bicocca, using a standard protocol described in^72^.

#### Sample analysis

Different cell conditions were examined: glial cells without treatment/induction (control, CTRL), glia cells induced with 10 ng/ml LPS (LPS), glia cells treated with 100 nM SAHA or 5 μM SAHA (SAHA100 or SAHA5), glia cells treated with 100 nM SAHA or 5 μM SAHA with a simultaneous LPS induction (LPS_SAHA100 or LPS_SAHA5).

For each condition, three samples were considered for the analysis of gene expression by AMDA microarray technology^72^.

#### Microarray comparisons and DEG analysis

Differentially expressed genes (DEGs) were identified comparing gene expression profiles of glial cells treated with 100 nM SAHA or 5 μM SAHA in the presence or the absence of the inflammatory stimulus. Comparisons are reported in:

- Table S1 (Supplementary Materials), LPS_SAHA100 vs LPS; LPS_SAHA5 *vs* LPS,
- Table S2 (Supplementary Materials), SAHA100 vs CTRL; SAHA5 *vs* CTRL,
- Venn diagrams (Fig. S5), SAHA100_CTRL vs SAHA100_LPS, SAHA5_CTRL *vs* SAHA5_LPS).

This multivariate analysis aims to reveal relationships between genes in the different treatment paradigms considered.

The identification of DEGs was addressed using Linear Models for Microarray Data (LIMMA). For the detection, linear modeling approach and empirical Bayes methods together with false discovery rate correction of the p-value (Benjamini-Hochberg) were used to moderate the standard errors. All the requested comparisons were performed selecting DEG with a threshold p Value of 0.01 as the criterion for statistical significance with a fold-change threshold of 1.

### Analysis of data and canonical pathway enrichment analyses

DEGs significantly (log 2-Fold Change and P values) up- or downregulated genes were associated with canonical pathways. A functional annotation of DEG is performed based on a subset of the annotation provided by the Bioconductor project (www.bioconductor.org)^72^.

Functional annotation: Canonical pathway enrichment analyses were performed using Kyoto encyclopedia of genes and genome (KEGG) pathways as well as Gene Ontology (GO) enrichment analyses (www.genome.jp/kegg, www.geneontology.org respectively). The most representative functional annotations for DEGs from each experimental condition were identified and only the top 40 statistically most significant annotation terms are reported. If p-values were lower than 10^–5^, the enrichment was considered significant^72^.

Enrichment analysis: Only pathways represented with at least 2 DEGs were ranked according to their relevance. Based on this ranking only the first 40 (or less whenever this number was not reached) were reported. If at least two experimental conditions were analyzed, a functional summary reporting the enrichment p-values less than 0.05 (log10 of p-value) for the different annotation terms and their similarity is provided ^72^.

Tables embedded in the Supplementary Information contain lists of the relevant annotation terms for each cluster and for each annotation source and the functional p value enrichment.

### Statistical analysis

All experiments were performed on at least two different primary cell preparations. Data are expressed as mean ± SEM. Data were analyzed using GraphPad Prism 9 edition (GraphPad Software, La Jolla, CA, USA). Statistical differences were determined using analysis of variance followed by multiple comparison tests, as indicated in the legends. Effects were designated significant when *p* < 0.05.

## Supplementary Information

Supplementary Information 1.

Supplementary Information 2.

## Data Availability

The datasets used and/or analysed during the current study available from the corresponding authors on reasonable request.
