# Supplementary figures and images for: Dose-dependent dual effects of HDAC inhibitors on glial inflammatory response
Source: Sci Rep. 2025 Apr 10;15:12262. doi: 10.1038/s41598-025-96241-x (PMC11986048; doi:10.1038/s41598-025-96241-x)

Figure S1

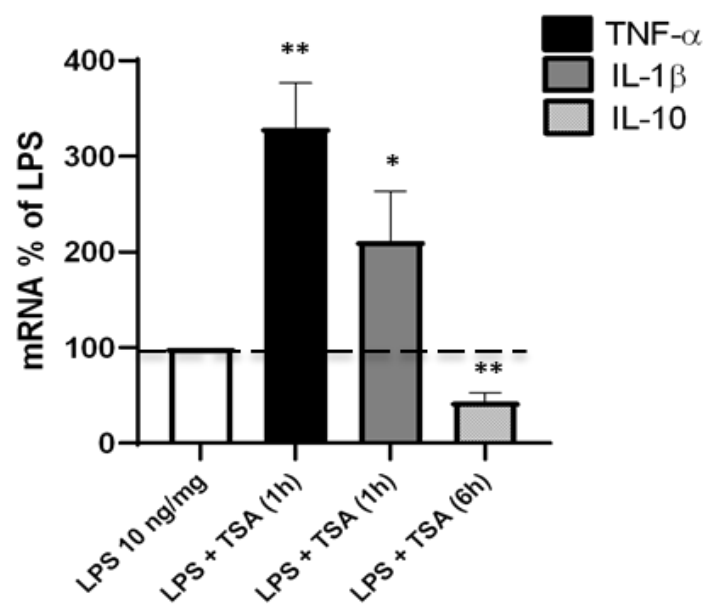

Figure S2

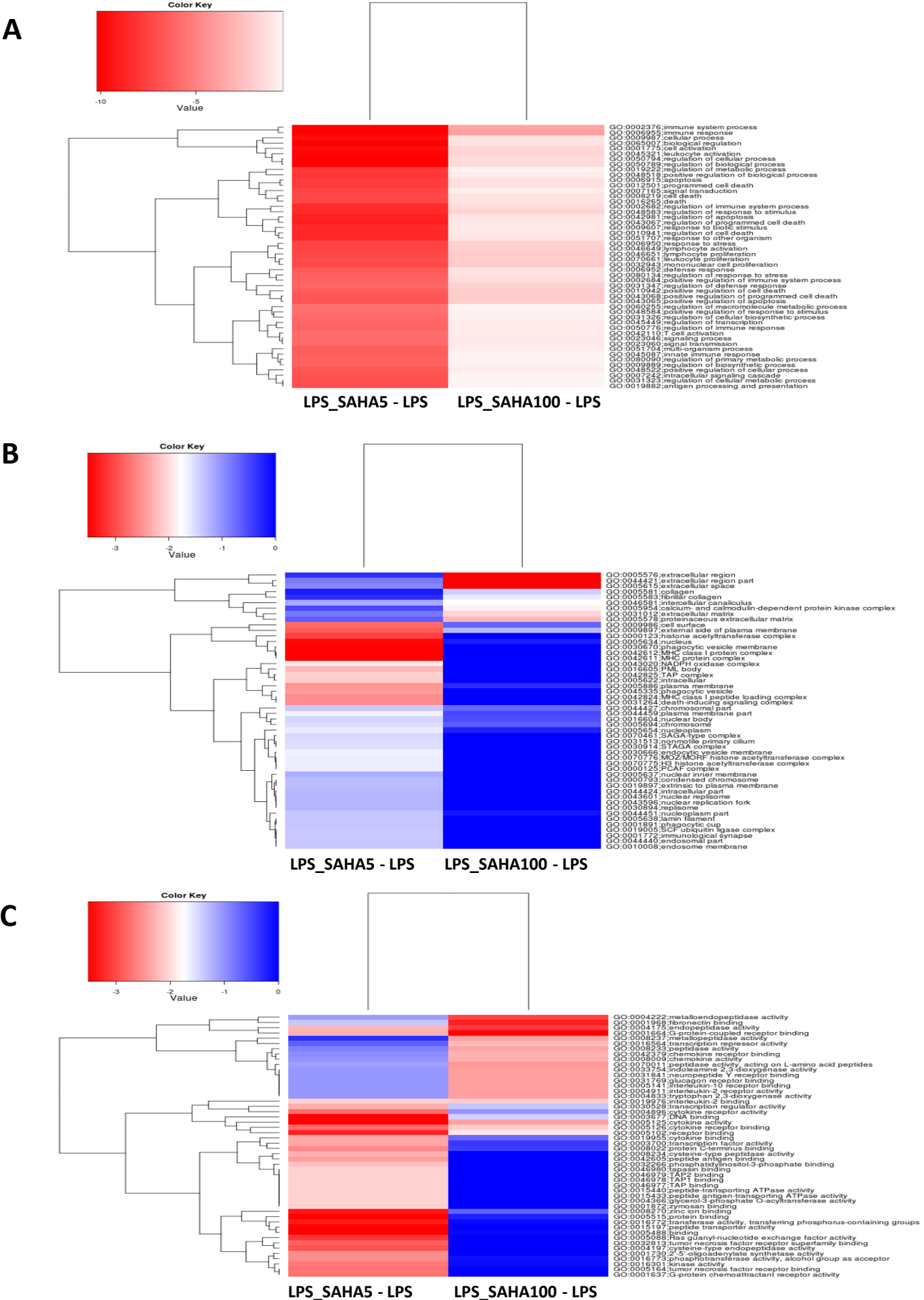

Figure S3

A *Gene expression profile*

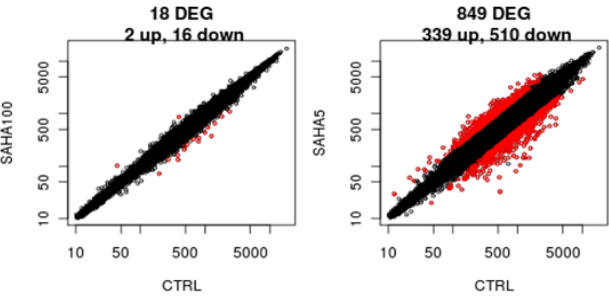

B *Pathway enrichment*

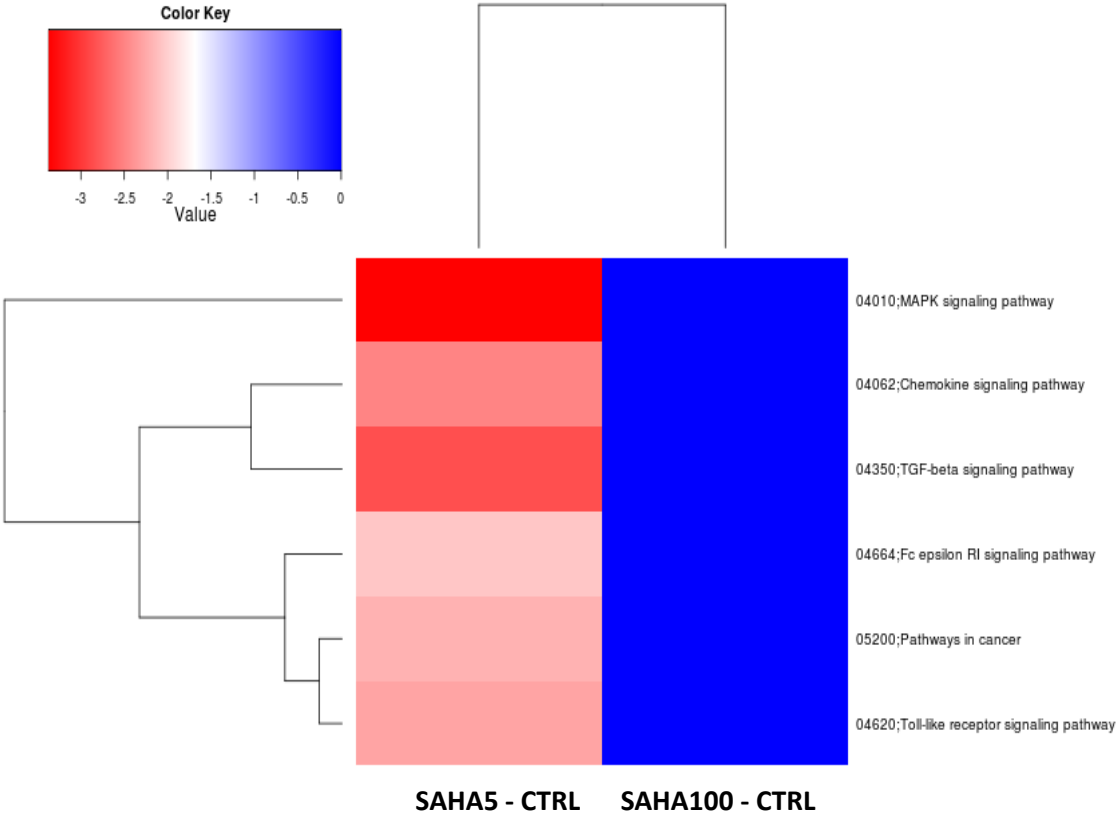

**A**

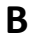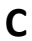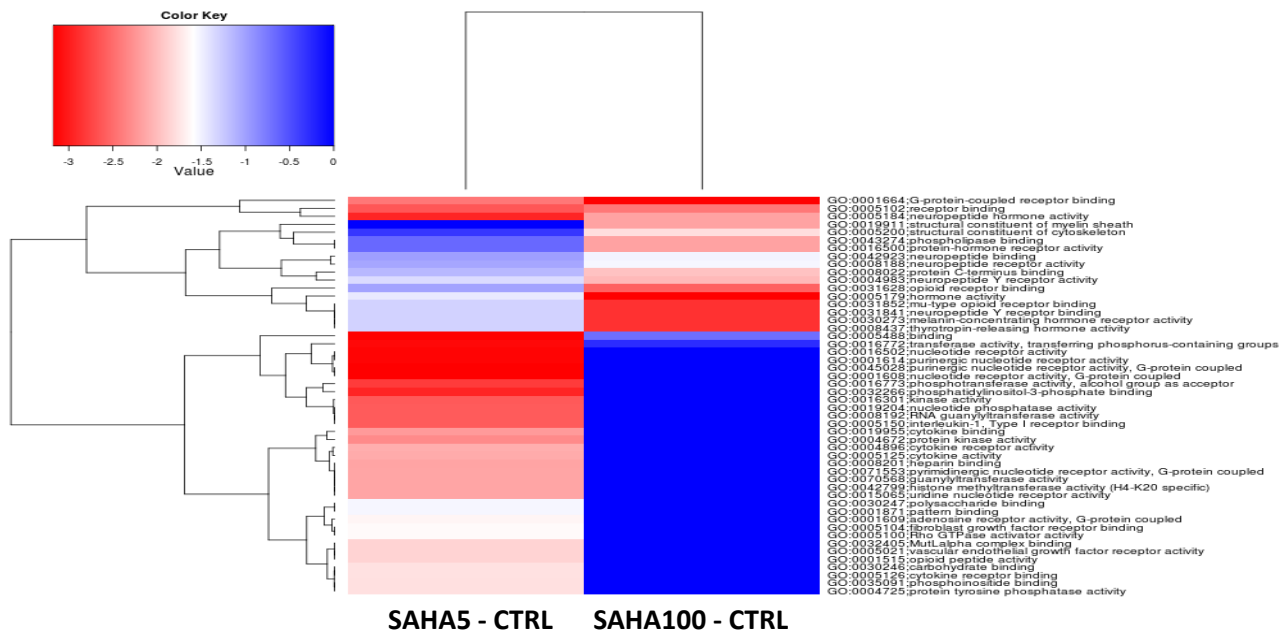

Figure S5

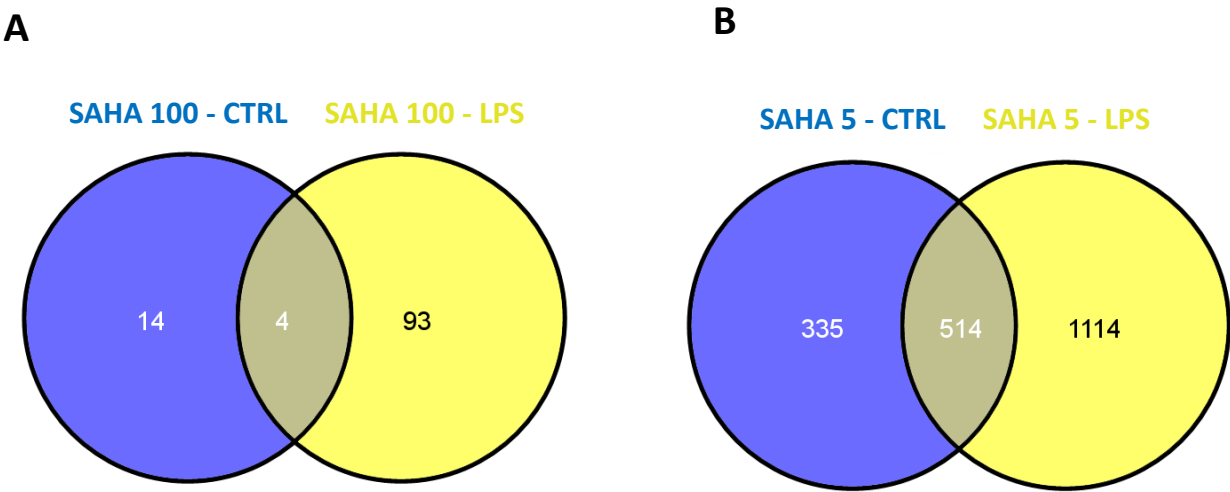

Supplement: Supplementary file 1 — Supplementary Information 1. [file 41598_2025_96241_MOESM1_ESM.pdf]
