# Supplementary material for: Dose-dependent dual effects of HDAC inhibitors on glial inflammatory response
Source: Sci Rep. 2025 Apr 10;15:12262. doi: 10.1038/s41598-025-96241-x (PMC11986048; doi:10.1038/s41598-025-96241-x)
Supplement: Supplementary file 2 — Supplementary Information 2. [file 41598_2025_96241_MOESM2_ESM.pdf]

## SUPPLEMENTARY FIGURES

**Figure S1.** Short (1 and 6 hours) exposure of glial cells to 10 ng/ml LPS in the absence or simultaneous presence of 10 nM TSA to evaluate mRNA levels of TNF- α (black bars), IL - 1β (grey bars) and IL - 10 (stripped bars) by RT-PCR.

**Figure S2. GO functional summary** for the comparison LPS_SAHA (100 or 5) - LPS. **A)** Biological Process (BP) **B)** Molecular Function (MF) **C)** Cellular Components (CC), reporting the enrichment p-values less than 0.05 (log10 of p-value).

**Figure S3. A) Scatter plots of log2 intensity values of DEGS.** Total number of DEGs (red dots) identified for glial cells treated with 100 nM or 5 uM SAHA compared to control (CTRL) condition.
DEGs identified with the Limma algorithm with a threshold p-value of 0.01. On the y-axis are reported the means of the replicates of the treatment conditions and on the x-axis the mean across the replicates of the reference condition (CTRL). **B) Pathway enrichment analysis.** KEGG functional summary reporting the enrichment p-values less than 0.05 (log10 of p-value) for the different annotation terms and their similarity for the comparisons SAHA100 - CTRL and SAHA5 - CTRL. The color scheme is based on a simple scale painting pathways (blu down – and red up- regulated) by automatic analysis using Basic Analysis.

**Figure S4. GO functional summary** for the comparison 100 nM or 5 uM SAHA - CTRL. **A)** Biological Process (BP) **B)** Molecular Function (MF) **C)** Cellular Components (CC), reporting the enrichment p-values less than 0.05 (log10 of p-value).

**Figure S5.** Treatment effects on gene expression in control cells and LPS-induced cells. Venn diagrams illustrate the overlap in gene expression modifications between comparisons **A)** *SAHA 100 - CTRL and SAHA 100 - LPS*, **B)** *SAHA 5 - CTRL and SAHA 5 - LPS* and their intersections.

## SUPPLEMENTARY TABLES:

**Table S1.** DEGs members detected as significantly expressed for the condition LPS_SAHA100 - LPS and LPS_SAHA5 - LPS. Color schema follows the statistic of differentially expressed genes (blue stands for down – and red for up-regulated genes, NA- not detected).

**Table S2**. DEGs members detected as significantly expressed for the condition SAHA100 – CTRL and SAHA5 - CTRL. Color schema follows the statistic of differentially expressed genes (blue stands for down – and red for up-regulated genes, NA- not detected).

**Table S3**. DEGs members detected as significantly expressed in the intersections of the Venn Diagram between SAHA 5 - CTRL and SAHA5 - LPS as well as SAHA 100 - CTRL and SAHA100 - LPS. Color schema follows the statistic of differentially expressed genes (blue stands for down – and red for up-regulated genes, NA- not detected).
